# Bombesin receptor-activated protein homolog deficiency altered the pattern of pathological changes of psoriasis - like skin lesion in mice

**DOI:** 10.7150/ijms.89492

**Published:** 2024-01-01

**Authors:** Jiaoyun Zheng, Hui Wang, Jie Wang, Zhi Peng, Xueping Yao, Horst Christian Weber, Xiaoqun Qin, Yang Xiang, Chi Liu, Ming Ji, Huijun Liu, Xiangping Qu

**Affiliations:** 1 Department of physiology, School of Basic Medical Science, Central South University, Changsha 410008, Hunan, China.; 2 Department of pathology, The Second Xiangya Hospital, Central South University, China.; 3 Functional Center, School of Basic Medical Sciences, Xinjiang Medical University, China.; 4 Boston University School of Medicine, Section of Gastroenterology, and Department of Pathology and Laboratory Medicine, Boston, MA 02118, USA.

**Keywords:** BRAP, TSLP, keratinocyte, imiquimod, Psoriasis

## Abstract

This study investigated the potential role of the mouse homolog of bombesin receptor-activated protein (BRAP) in imiquimod (IMQ) induced psoriasis - like skin inflammation. The expression of both human BRAP, encoded by *C6orf89*, and its mouse homolog, encoded by *BC004004*, has been found to be expressed abundantly in the keratinocytes. *BC004004* knockout mice (*BC004004^-/-^*) were topically treated with IMQ daily for 7 days to test whether they were more vulnerable to psoriasis - like inflammation. We found that those mice exhibited an altered pattern of inflammation process compared to isogenic wild type control mice (*BC004004^+/+^*). *BC004004^-/-^* mice developed skin lesions with earlier and more acute onset, as well as a quicker remission. The cytokines related to pathogenesis of psoriasis also exhibited different expression patterns in IMQ treated *BC004004^-/-^* mice. On day 4 of IMQ treatment, *BC004004^-/-^* mice exhibited a higher expression level of IL-17A compared to *BC004004^+/+^* mice, suggesting a more robust activation of Th17 cells in the knockout mice. The serum level of thymic stromal lymphopoietin (TSLP), one of the keratinocyte derived cytokines, was also increased in *BC004004^-/-^* mice and reached its peak on day 4. Knockdown of BRAP in cultured human keratinocyte-derived HaCaT cells by siRNA silencing led to increased release of TSLP. Our data suggest that the elevated of level of TSLP released from keratinocytes due to BRAP deficiency might mediate the crosstalk between the epidermal cells and immune cells and thereby contributing to the altered pathological changes observed in psoriasis - like skin lesion in knockout mice.

## 1. Introduction

As the outermost covering of the body, the skin protects against injury and infection not only by serving as a physical barrier but also by regulating immune responses and participating in maintaining homeostasis^1^. The most prominent cellular constitutes among the skin are keratinocytes, which play a crucial role in maintaining the integrity of the epidermis and sensing environmental stressors^2^. In addition, keratinocytes are involved in skin immune responses by the secretion of various cytokines which mediate the crosstalk between epidermis and immune cells^3^-^5^. Disturbance of biological function of keratinocytes may lead to pathological changes of skin, which are often associated with distinct types of immune responses. Growing evidence has shown that activated keratinocytes may play a pivotal role in pathogenesis of psoriasis vulgaris, which is one of the most common chronic inflammatory skin lesions^6^, ^7^. Psoriasis is characterized by increased proliferation of keratinocytes, thickening of epidermis and infiltration of immune cells in both epidermis and dermis. The communication between different cell types, including dendritic cells (DCs), T cells and keratinocytes, was shown to be involved in the development of disease, whilst the pathophysiology of this disease is still not fully understood^8^-^11^. Our group found that human bombesin-receptor activated protein (BRAP), encoded by *C6orf89* gene, was expressed in keratinocytes of normal skin and lesions of psoriasis. The mouse homolog of BRAP, which is encoded by *BC004004* gene and shares 83% similarity to the human BRAP, was also expressed in mouse skin. In our previous study, we investigated the function of BRAP and its mouse homolog by utilizing a gene knockout mouse model *BC004004^-/-^*, which led to several distinct phenotypes 12-14. Compared with their isogenic wild type control, *BC004004^-/-^* mice exhibited attenuation of injury induced fibrosis in both lungs 12 and kidneys (data not published), exacerbation of stress induced behavioral changes^13^ and abnormal cilia formation in trachea epithelial cells^14^. The underlying cellular and molecular mechanisms remain largely unknown, but our studies provide evidence for altered behaviors of several cell types including lung fibroblasts, tubular epithelial cells and neurons due to lack of BRAP expression. To explore the role of BRAP or its mouse homolog in skin diseases we established the psoriasis model by tropical application of imiquimod (IMQ) using *BC004004^-/-^* mice and the control *BC00400^+/+^* mice. Lack of BRAP homolog led to changes of pathological progress of skin lesions and altered expression patterns of associated inflammatory cytokines. Furthermore, serum level of thymic stromal lymphopoietin (TSLP), a cytokine derived from keratinocytes^15^, was also elevated in gene knockout mice after IMQ application, and knock down of BRAP in cultured human HaCaT keratinocytes also caused increased secretion of TSLP. Those data suggest lack of BRAP expression might affect communication between keratinocytes and other immune cells during the pathological process of psoriasis via the secretion of TSLP by keratinocytes.

## 2. Materials and methods

### 2.1 Animals

All animal use was approved by Xiangya animal care and use committee at Central South University (XMSB-2022-0003). All clinical samples were obtained from the department of pathology at the second xiangya hospital, and human experiments were conducted in accordance with the standard operating guidelines of the Institutional Animal Care and Research Advisory Committee of Central South University, Changsha, China (No: 2022-KT118). Seven weeks old female *BC004004^-/-^* mice, in which BC004004 gene was disrupted by CRISPR/Cas9-mediated genome engineering technique 12, and their isogenic control C57BL/6 mice (*BC004004^+/+^*) were used in the study.

### 2.2 Mouse model of IMQ induced psoriasis-like skin inflammation

Mice were randomly divided into IMQ treatment groups (*BC004004^+/+^* + IMQ and *BC004004^-/-^* +IMQ) and control groups with a control cream (*BC004004^+/+^* + Vaseline and *BC004004^-/-^* + Vaseline). IMQ treatment was performed as described in previous studies 16, 17. Briefly, the back side area of the mice anesthetized with isoflurane was shaved carefully without damage to the skin on the day previous to IMQ application. From the next day (Day 1) the IMQ treatment groups received a daily topical application of 62.5 mg of commercially available imiquimod 5% cream (Mingxinlidi™, Sichuan Med-Shine Pharmaceutical) on their shaved back for consecutive 7 days. The control groups received a daily topical administration control vehicle cream (Vaseline cream; Unilever). The application of Mingxinlidi™ cream represents a daily dose of 3.125 mg of imiquimod. The skin inflammation was scored according to a murine Psoriasis Area and Severity Index (PASI) score table 16, 17. Briefly, redness was scored on the following scale: 0 for none (no redness), 1 for light red, 2 for red not dark, 3 for dark red, 4 for extremely red. Scaling was scored on the following scale: 0 for none (no scaling), 1 for thin scales and limited surface area, 2 for thicker (mild) scales and modest surface area, 3 for thicker scales, rough and larger surface area, 4 for very robust scales, vast surface area. And thickening was scored on a scale from 0 to 4: 0, none; 1, slight; 2, moderate; 3, marked; 4, very marked.

### 2.3 Histological analysis of skin tissues

Mice were sacrificed by an intraperitoneal injection of lethal dose of phenobarbital sodium. Skin tissues were fixed in 4% paraformaldehyde overnight and then were embedded in paraffin and the subsequent Hematoxylin and eosin (H&E) staining, immunohistochemistry (IHC) and immunofluorescence analysis were performed as described previously 12. The following antibodies and their corresponding dilutions were used: Myeloperoxidase (MPO) (266-6K1) (Santa Cruz, cat: sc-52707, 1:1,00); F4/80 (Servicebio, cat: GB113373-100, 1:2,00), CD3 (Santa Cruz, cat: sc-20047, 1:1,00), IL-17 (Affinity, cat: DF6127, 1:200), IL-17 (Santa Cruz, cat: sc-374218, 1:200), CK5 (Affinity, cat: AF5479, 1:200), CD11C (Bioss, cat:bs-2508, 1:200), TSLP (Affinity, cat:DF8077, 1:200).

The double immunofluorescence analysis was carried out according to the previous method 12. Briefly, the tissue sections were visualized using either Alexor Fluor 488 AffiniPure donkey anti-rabbit fluorescent secondary antibody (Jackson ImmunoResearch, cat: 711-545-152, 1:200), or Cy^TM^3 AffiniPure donkey anti-mouse fluorescent secondary antibody (Jackson ImmunoResearch, Cat: 715-165-150, 1:200). The nuclei were counterstained with DAPI in immunofluorescence at a dilution of 1:10 (Servicebio, cat: G1012).

### 2.4 Cell culture

HaCaT, an immortalized human keratinocyte cell line, was purchased from National Collection of Authenticated Cell Cultures^18^, ^19^ and cultured at 37°C with 5% CO_2_ in DMEM (Invitrogen, cat: 11960-044) containing 10% fetal bovine serum, 1% Gibco™ GlutaMAX™ (Invitrogen, cat: 35050061) and 1% sodium pyruvate solution (100nM) (Invitrogen, cat: 11360070). The expression of human BRAP was down-regulated by gene silencing mediated by Stealth RNAi^TM^ siRNAs targeting *C6orf89* gene (ThermoFisher Scientific, siRNA ID: HSS137527, HSS137528 and HSS137529) which was transfected into cells by Lipofectamine™ 3000 (ThermoFisher Scientific, cat: L3000008) according to the manufacturer's protocol.

### 2.5 Western blot analysis

Total protein was extracted from skin tissues or from cultured cells and then subjected for Western blot as described previously 12. The following antibodies and their corresponding dilutions were used: BRAP (Abcam, cat: ab181073; 1:2,000), IL-23 (Santa Cruz, cat: sc-271279,1:1,000;), IL-1β (Santa Cruz, cat: 12742,1:1,000), IL-10 (Santa Cruz, cat: 8438, 1:1,000), IL-17 (Affinity, cat: DF6127, 1:2,000), IL-17 (Santa Cruz, cat: sc-374218, 1:1,000), TNF-α (Santa Cruz, cat: sc-52746, 1:1,000), CK5 (Affinity, cat: AF5479, 1:2,000), β-Actin (Santa Cruz, cat: sc-47778, 1:5,000), CK14 (Santa Cruz, cat: sc-53253, 1:1,000), TGF-β1 (Abcam, cat: ab64715, 1:2,000), HRP-conjugated goat anti-Mouse IgG (H+L) (Proteintech, cat: SA00001-1, 1:5,000), HRP-conjugated goat anti-rabbit IgG(H+L) (SouthernBiotech, cat: 4050-05; 1: 5,000).

### 2.6 Enzyme-linked immunosorbent assay (ELISA)

The contents of IL-17, IL-23 and TSLP in mouse serum were quantified by commercially available ELISA kits according to the manufacturers' instructions. The following ELISA kits were used: mouse IL-17A/F ELISA MAX™ Deluxe Set (BioLegend, cat: 436204); mouse IL-23 ELISA MAX™ Deluxe Set (BioLegend, cat: 433704) and mouse TSLP ELISA MAX™ Deluxe Set (BioLegend, cat: 434104). 20 μL of serum was diluted with 80μL of 1X Assay Diluent A and then was added to one well of a 96-well plate for the assay. 100 μL of culture media of HaCaT cells was added to one well of a 96-well plate for measurement of TSLP or TGF-β1 level using either human TSLP ELISA MAX™ Deluxe Set (BioLegend, cat: 434204) or TGF-β1 ELISA MAX™ Deluxe Set (Elabscience, cat: E-EL-0162c).

### 2.7 Statistical analyses

Statistical analyses were conducted on GraphPad Prism 7. Results are expressed as the mean of independent experiments ± SD. Two-way ANOVA was used to analyze the effects of two factors (IMQ treatment or genotype) on body weights and PASI score. It was performed according to the methods described previously 12. One-way ANOVA was used for all the other data except the effect of siRNA silencing of BRAP in HaCaT cells on TSLP levels in culture media, which was analyzed using a two-tailed unpaired Student's t-test. A *P* value less than 0.05 was considered statistically significant.

## 3. Results

### 3.1 The expression of BRAP and its homolog in skin tissues

**Figure 1:**
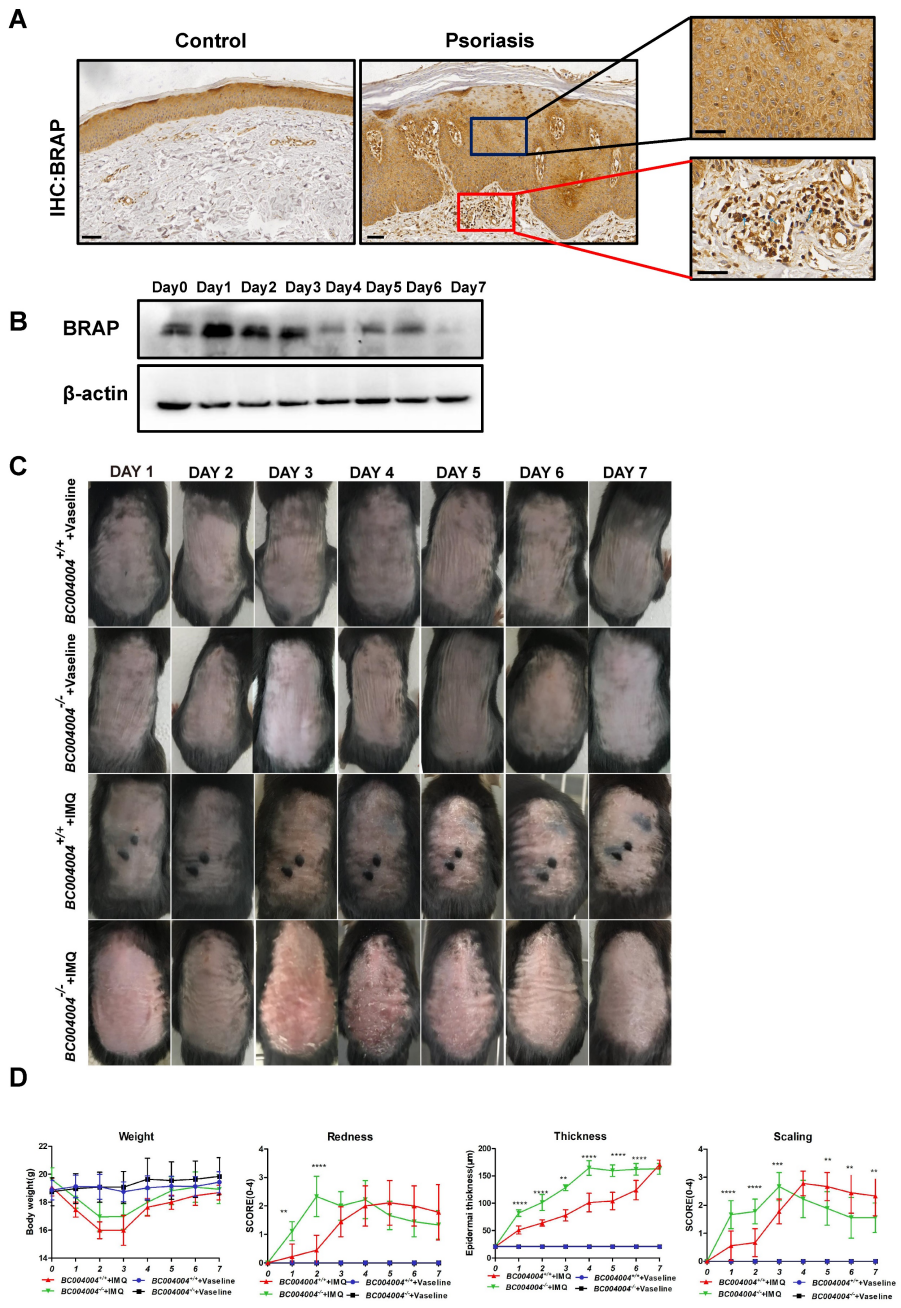
** The expression of BRAP and its mouse homolog in skin tissues and the skin injury induced by IMQ in both *BC004004^-/-^* mice and their wild type control mice.** (A) The expression of BRAP in both normal human skin and the skin of patient with psoriasis by immunohistochemistry analysis of tissue samples with anti-BRAP antibody. BRAP is expressed in the epidermis of both normal skin tissue and skin tissue with psoriasis (magnification: 100×(scale bar = 200 μm) and 400×(scale bar = 50 μm)). (B) BRAP homologous protein was present in skin tissues as revealed by Western blotting in wild-type control mice *BC004004^+/+^* with imiquimod (IMQ) treatment for 7 day. The total protein extract from one wild type mice skin representing each day of IMQ treatment was loaded in one well. The expression of β-actin from the same sample was used as a loading control. (C) Representative photographs of psoriasis-like lesions on back skin of *BC004004^+/+^* mice and *BC004004^-/-^* mice after 7 days of IMQ treatment. Vaseline treatment was applied on control groups as a vehicle control. (D) Daily body weights throughout the 7-day IMQ treatment were shown in the upper left panel. The characteristics of skin lesion were depicted as redness, scaling scores and thickness of back skin and scored according to a murine Psoriasis Area and Severity Index (PASI) score table. Data are presented as mean ± SD; n = 9 in each group. *p<0.05, **p<0.01, ***p<0.001, ****p<0.0001.

As illustrated by IHC analysis using an antibody against human BRAP (EPR13621, cat: ab181073, Abcam), BRAP was expressed in various cells from both epidermis and dermis. BRAP was moderately expressed in keratinized stratified squamous epithelium, as well as in dermal blood vessels (Figure 1A). The IHC staining of tissue samples from psoriasis patients also showed that BRAP was present in epidermis with increased layers of keratinocytes (Figure 1A). Interstitial cells of dermis connective tissue and the infiltrated inflammatory cells exhibited weak expression of BRAP. As described in our previous study, this antibody is not suitable for immunostaining of mouse tissues. But it gives specific signal of BRAP homolog in western blot analysis when using total protein extracts of tissues from mice. We detected BRAP homolog expression levels in skin tissues of wild type mice (*BC004004^+/+^*) during IMQ treatment process. As shown in Figure 1B, the expression of BRAP homolog increased on the first day of IMQ application (Day 1) and then decreased continually thereafter. The results of western blot analysis using protein extracts from skin tissues of *BC004004^+/+^* mice on Day 1, Day 4 and Day7 of IMQ applications also showed the same expression tendency (Figure 3B).

### 3.2 *BC004004^-/-^* mice exhibited an altered pattern of IMQ induced skin inflammation process

Figure 1C shows the appearance of back skin over successive days during IMQ application procedure. Topical IMQ application led to apparent skin lesions such as redness, scaling and epidermal thickening in both wild type mice (*BC004004^+/+^*) and gene knockout mice (*BC004004^-/-^*) compared with vehicle control groups. All the observations were made 24 hours after the IMQ applications. For the illustrations in Figures, Day 0 indicates that the measurement was made before the first IMQ application. Then IMQ applications were made for the following successive 7 days with 24 hours internal between each application. Day 1 means the measurement was made 24 hours after the first IMQ application and Day 7 means the measurement was made 24 hours after the last IMQ application. The redness and scaling seems to be most pronounced on Day 2 and 3 in *BC004004^-/-^*+ IMQ group. In wild type mice IMQ led to most apparent skin changes on Day 4 and 5. In both wild type mice (*BC004004^+/+^*) and gene knockout mice (*BC004004^-/-^*) tropical IMQ application led to significant body weight loss on Day 2 and Day 3 as shown in Figure 1D. From Day 4 onward, the body weight of both groups increased gradually and then there was no significant difference on body weight changes between those two groups. The psoriatic like inflammation were evaluated by scoring the skin redness (erythema) and scaling according to a PASI score table 16, 17. Compared with wild type control, the skin inflammation of the gene knockout mice has an earlier and more acute onset since all the scores of redness and scaling of skin increased more significantly from Day 1 of IMQ application (Figure 1D). The peak of redness score was reached on Day 3 in *BC004004^-/-^*. On Day 6 and Day 7 of IMQ application the intensity of skin redness in *BC004004^-/-^* mice was similar to that of wild type control mice. The scaling scores of *BC004004^-/-^* mice were much higher than those of *BC004004^+/+^* mice on Day 1, 2 and 3 of IMQ application. Then on Day 4, 5, 6 and 7 of IMQ the scaling scores of those knockout mice dropped and were much lower than those of *BC004004^+/+^* mice. The epidermal thickening was assessed by measuring the thickness of epidermis from skin tissue sections under the microscope. IMQ application led to more significant increases on epidermal thickness in *BC004004^-/-^* mice compared with wild type control mice for all the time points (Day 1 through Day 7).

**Figure 2:**
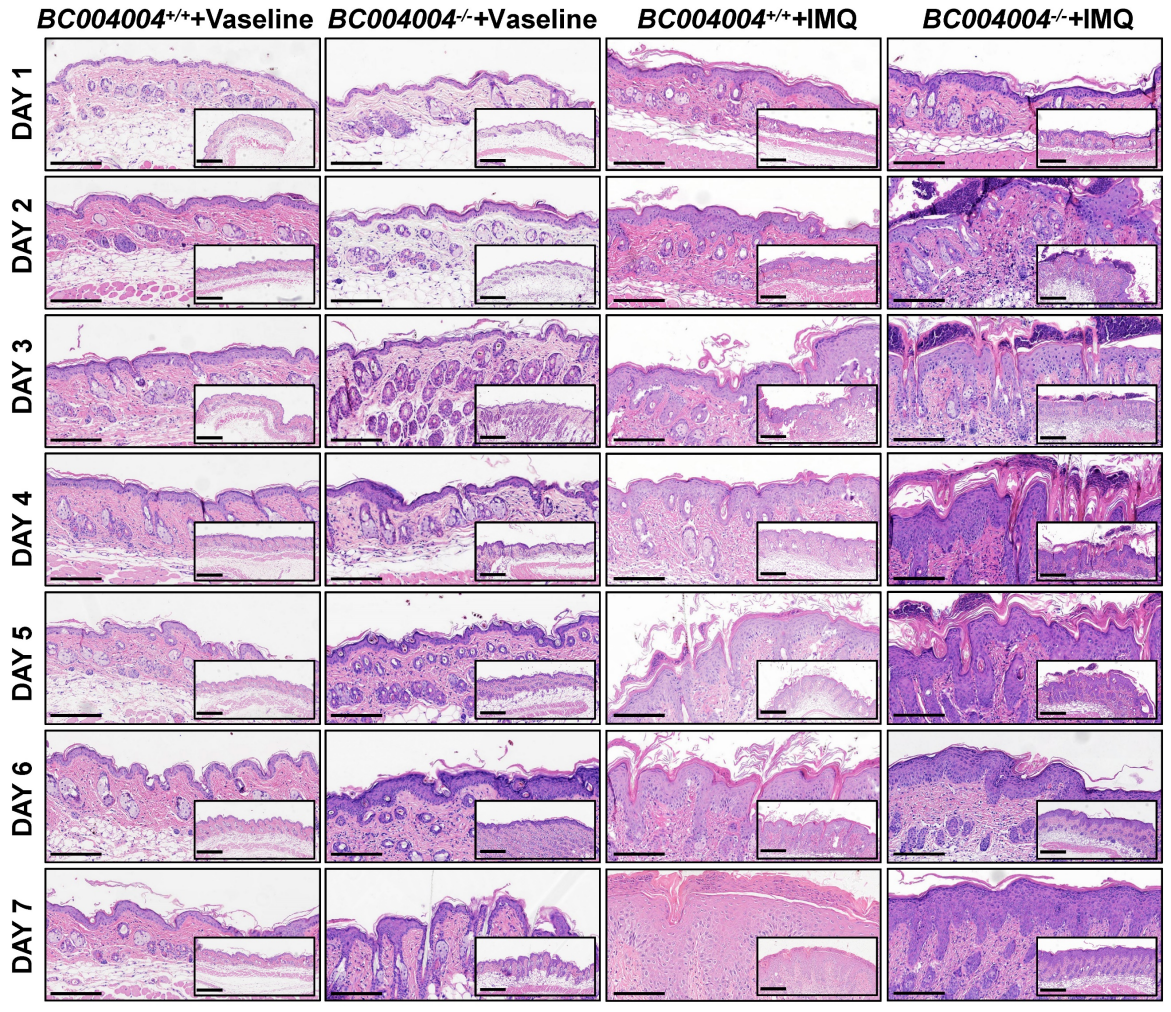
Representative histological changes of skin tissue sections from both *BC004004^-/-^* mice and their wild-type control mice after IMQ treatment. Original images were magnified at 100×(scale bar = 200μm) and 200×(scale bar = 100μm).

Hematoxylin-eosin (H&E) staining of skin sections showed psoriasiform epidermal hyperplasia after IMQ treatment in both *BC004004^+/+^* mice and *BC004004^-/-^* mice, which indicates that IMQ treatment altered keratinocyte proliferation and differentiation. As shown in Figure 2, *BC004004^-/-^* mice exhibited acute onset of inflammatory lesions including epidermal thickening, parakeratosis, hyperkeratosis, acanthosis and Munro's microabscesses after the first IMQ application (Day 1). The above skin inflammatory responses became most severe on Day 3 and 4 after IMQ application in *BC004004^-/-^* mice. On Day 4 infiltration of inflammatory cells and increased number of capillaries and dilated capillaries within dermis could also be seen in skin sections from IMQ treated *BC004004^-/-^* mice. Those symptoms became alleviated on Day 5 and only epidermal thickening and hyperkeratosis could be found on Day 6 and 7. Compared with gene knockout mice, the wild type control mice presented a delayed onset and more prolonged skin inflammation. Epidermal thickening, parakeratosis, hyperkeratosis and acanthosis could be found in skin from control mice on Day 2 and Munro's microabscesses could be found on Day 4. Those IMQ-induced inflammatory symptoms from wild type control mice were relatively milder and more sustained compared with those from *BC004004^-/-^* mice. On Day 7 Munro's microabscesses were still present in skin from IMQ treated wild type control mice while they were absent from *BC004004^-/-^* mice.

Taken together, *BC004004^-/-^* mice exhibited more acute onset of inflammation triggered by IMQ and then the inflammatory symptoms began to be alleviated after 4 days. At the end of IMQ procedure the skin lesions on gene knockout mice were even less than those of wild type control mice.

### 3.3 BRAP homolog deficiency led to alteration of cytokine expression patterns during the topical IMQ treatment process

**Figure 3:**
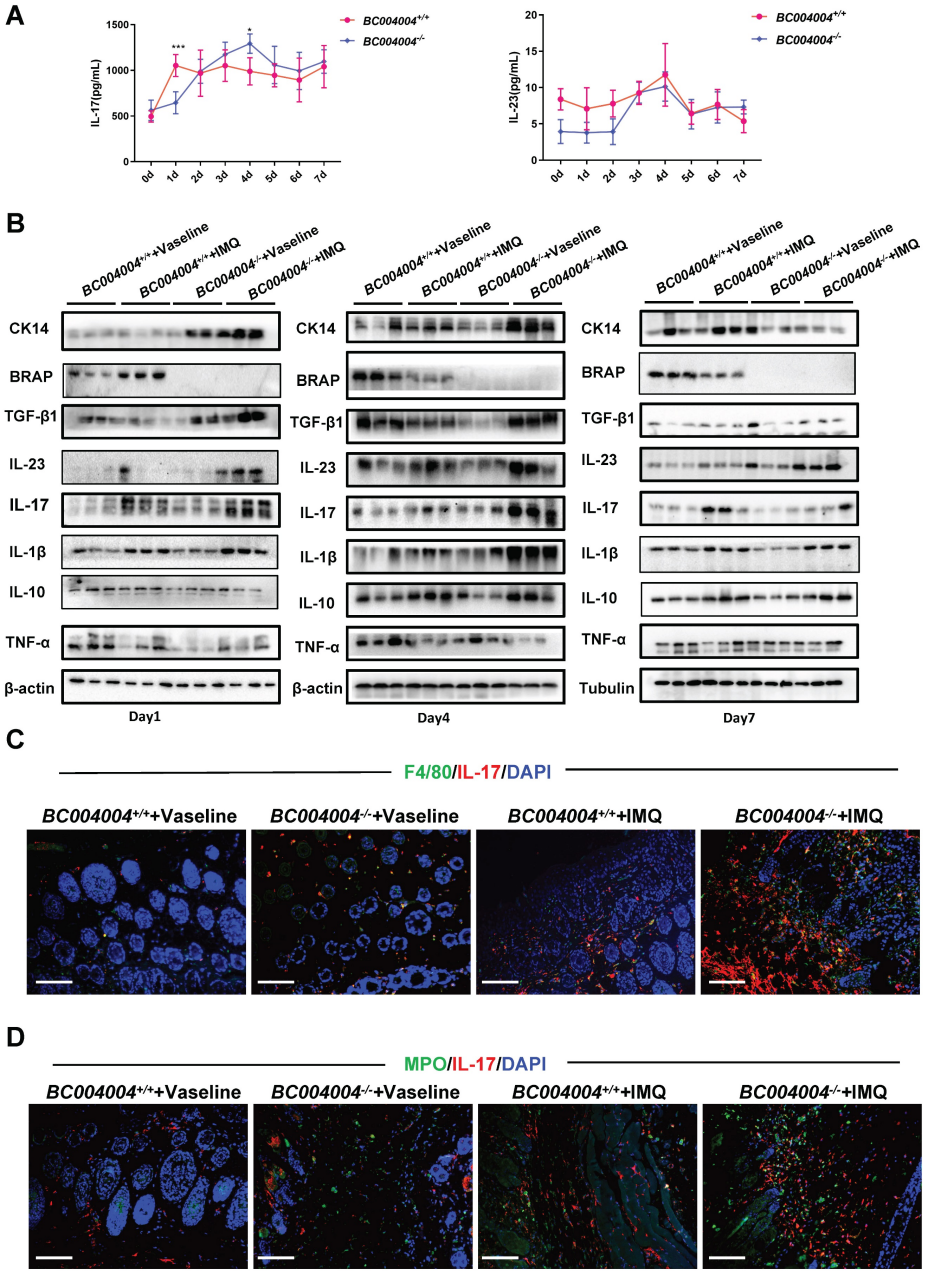
** The different expression patterns of the cytokines related to pathogenesis of psoriasis in IMQ treated *BC004004^-/-^* mice.** (A) Daily serum levels of IL-17 and IL-23 during the 7-day IMQ treatment as determined by ELISA. (B) The protein levels of CK14 and different cytokines in skin tissues from 3 mice were assessed by western blotting analysis on Day 1, 4 and 7 after IMQ treatments. (C) Representative images of double immunofluorescence staining in skin tissue sections from IMQ treated mice. Immunostaining with IL-17 antibody was shown as red fluorescence. Immunostaining using antibodies against F4/80 or MPO was shown as green fluorescence. Nuclei of the cells were localized by DAPI (blue). The orange signals in the merged picture indicate the co-distribution of IL-17 and either F4/80 or MPO in the tissue sections (magnification: 200×); scale bar = 100 μm.

Psoriasis is a chronic inflammatory disease involving complex immune responses. We measured some cytokines that have been shown to be critically involved in pathogenesis of psoriasis. The IL-23/Th17 axis was reported to play an important role in psoriasis [20, 21]and therefore the serum concentrations of the related cytokines IL-23 and IL-17 were measured after each application. As shown in Figure 3A, the serum concentration of IL-17 of wild type mice was higher than that of *BC004004^-/-^* mice after the first IMQ application (Day 1). Then the serum concentration of IL-17 of *BC004004^-/-^* mice gradually rose to the peak value on Day 4 and it was higher than that of wild type control on Day 4. There was no significant difference on serum concentrations of IL-23 between gene knockout mice and their wild type control mice during the overall IMQ treatment.

We considered the local cytokine levels within epidermis and dermis might play more important roles in regulating the behaviors of the cell with skin. Therefore we detected the expression levels of some cytokines, as well as CK14 and BRAP homolog using total protein extracts from skin tissues on Day 1, 4 and 7 by Western blot analysis. BRAP homolog expression increased on Day 1 in wild type mice and then decreased on Day 4 and Day 7. CK 14 is a type I acidic keratin that is present in mitotically active keratinocytes in the basal layer and can be used as a marker for keratinocytes which are responsible for the regeneration of stratified epithelial cells^22^. As shown in Figure 3B, after the first application (Day 1) there was a significant increase on the expression of CK14 in skin from* BC004004^-/-^* mice compared with wild type control. On Day 4 CK14 expression level in skin of gene knockout mice was still higher than that of wild type control. But on Day 7 CK14 expression from *BC004004^-/-^* mice was similar to that of *BC004004^+/+^* mice. Those data provide further evidence that *BC004004^-/-^* mice exhibited more acute onset of IMQ-induced keratinocytes proliferation.

After the first IMQ application (Day 1), besides CK14, the IMQ treated skin from *BC004004^-/-^* contained more TGF-β1 and IL-23 compared with that of *BC004004^+/+^* mice (Figure 3B, left panel). There were no difference on the contents of IL-17, IL-1β, IL-10 and TNF-α between* BC004004^+/+^* and *BC004004^-/-^* on Day 1. Then on Day 4 (after the fourth IMQ applications) there were more abundant IL-1β, TGF-β1 and IL-17 present in skin from *BC004004^-/-^*mice compared with wild type controls (Figure 3B, middle panel). On Day 4 the IL-23 content in skin from wild type controls also increased and was similar to that of gene knockout mice. 24 hours after the last IMQ application (Day 7) only IL-23 in skin of *BC004004^-/-^* mice was still more abundant compared with controls (Figure 3B, right panel). There were no significant changes on the contents of all the other cytokines between* BC004004^+/+^* and *BC004004^-/-^* mice.

### 3.4 Analysis of cells expressing IL-23 or IL-17 within mouse skin

**Figure 4:**
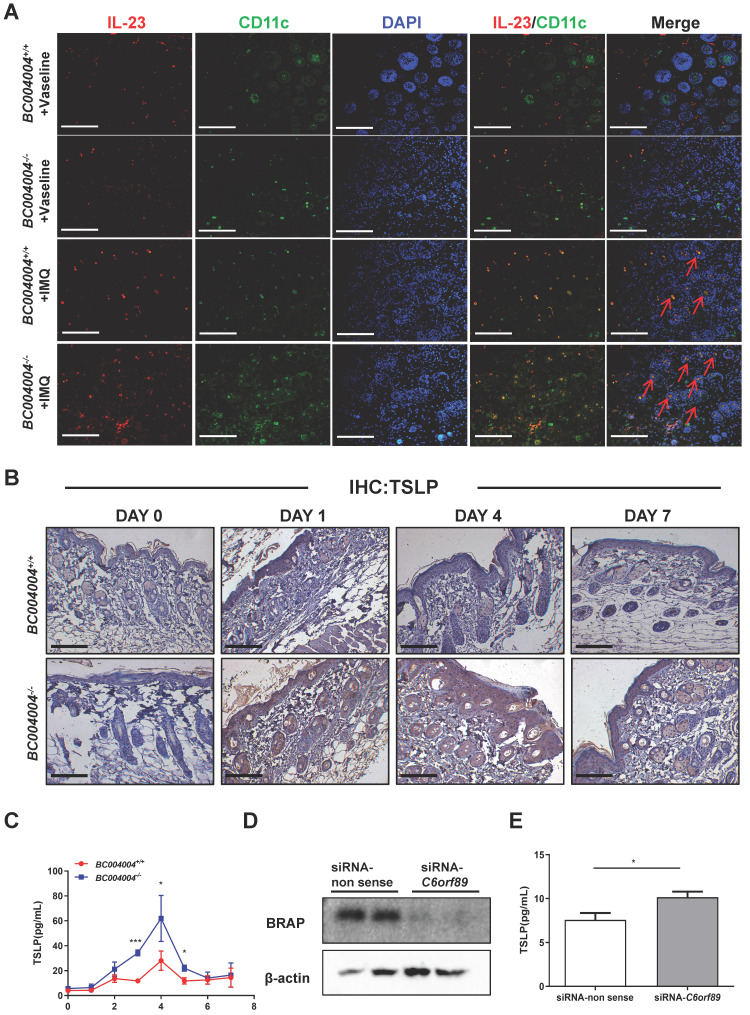
** TSLP expression was increased by down-regulation of BRAP or its mouse homolog.** (A) Double immunofluorescence staining by IL-23 and CD11c in skin tissue sections from IMQ treated mice (magnification: 200×); scale bar = 100 μm. Immunostaining with IL-23 antibody was shown as red fluorescence. Immunostaining using antibodies against CD11c was shown as green fluorescence. Nuclei of the cells were localized by DAPI (blue). The orange signals in the merged picture, some of which were shown by red arrows, indicate the co-distribution of IL-23 and CD11c in the cytoplasm of cells. (B) Immunohistochemistry analysis of skin tissue sections using TSLP antibody. Day 0 indicates that the samples were made before the first IMQ application. (C) Daily serum levels of TSLP during the 7-day IMQ treatment as determined by ELISA. (D) Western blot analysis of BRAP expression in HaCaT cells transfected with siRNA to silence *C6orf89* expression. (E) ELISA analysis of TSLP content in the culture media of HaCaT cells with *C6orf89* expression silenced by siRNA transfection.

The above examination of some important cytokines involved in pathogenesis of psoriasis within skin tissues reveals that BRAP homolog deficiency affects local cytokine contents and this might contribute to alterations of psoriasis-like inflammation process in *BC004004^-/-^* mice. IL-23 and IL-17 were shown as critical mediators for IMQ-induced psoriasis-like skin lesion in mice(17). We stained mouse skin tissue sections of Day 4 by double immunofluorescence staining in order to examine the types of cells within mouse skin that express IL-23 and IL-17. CD3, F4/80, CK5, MPO and CD11c were used as markers for T lymphocytes, macrophages, keratinocytes, neutrophils and dendritic cells, respectively. As shown by double immunofluorescence staining using anti-IL-17 antibody and anti-CD3 antibody, only a few green IL-17 fluorescence dots overlapped with red CD3 fluorescence which indicates the IL-17 was from a few lymphocytes in skin (Supplementary Figure 1). When tissue sections were stained with an anti-IL-17 antibody and an anti-F4/80 antibody, some red IL-17 immunofluorescence signals were found to be overlapped with green F4/80 fluorescence signals, indicating the co-distribution of IL-17 and F4/80 on some macrophages (Figure 3C). Double immunofluorescence staining also revealed a partially co-distribution of red IL-17 signals and green MPO signals (Figure 3D). MPO is mainly contained in neutrophils and also expressed in monocytes to a lesser degree^23^. Therefore, the green fluorescence signals in Figure 3D might be either from neutrophils or from monocytes. The partially co-distribution of red IL-17 signals and green MPO signals also suggests a possibility of neutrophils as a source for IL-17 production. However, most red IL-17 immunofluorescence did not overlap with either green CK5 immunofluorescence (Supplementary Figure 1), which indicates that IL-17 were not from keratinocytes. Skin tissue sections from Day 4 of IMQ were also stained with both anti-IL-23 and anti-CD11c antibodies. As shown in Figure 4A, a lot of red IL-23 immunofluorescence signals overlapped with green CD11c immunofluorescence signals and then showed orange signals in the merged picture. Since CD11c is a marker for dendritic cells, this result indicates that dendritic cells might be an important source of IL-23 within skin.

Taken together, the results of those double immunofluorescence assays suggest that some macrophages and a few lymphocytes within mouse skin could produce IL-17. And dendritic cells are an important source for IL-23 within skin tissues.

### 3.5 BRAP homolog deficiency increased TSLP production by keratinocytes

Since BRAP and its homolog were all found in human and mouse keratinocytes, we wondered whether deficiency of this protein affected the role of keratinocytes during IMQ treatment. TSLP is an IL-7 like cytokine that is predominantly produced by epithelial cells in tissues including lung, thymus and skin^15^. This cytokine mediates the cross-talk between epithelial cells and immune cells to regulate immune responses via its receptor TSLPR which was found on many immune cells including dendritic cells (DCs), lymphocytes and monocytes^24^, ^25^. As shown in Figure 4B, TSLP expression in epidermis of both *BC004004^+/+^* and *BC004004^-/-^* mice was pretty weak as revealed by immunostaining analysis of skin tissue sections using TSLP antibody. The IHC analysis of tissue sections on Day 1, 4 and 7 showed that IMQ treatment increased the content of TSLP within epidermis in both groups. However, TSLP in *BC004004^-/-^* mice was more abundant compared to that of *BC004004^+/+^* mice on Day 1, 4 and 7 of IMQ treatment. As shown in Figure 4C, the serum levels of TSLP of IMQ treated* BC004004^-/-^* mice gradually increased during treatment and peaked on Day 4. The serum TSLP levels in *BC004004^-/-^* mice were significantly higher than those of wild type controls. In addition, we assessed TSLP released by cultured HaCaT cells when BRAP expression was down-regulated in those cells. The expression of *C6orf89* gene was silenced in HaCaT cells using siRNA targeting *C6orf89*. The expression of BRAP protein was down-regulated as shown by western blot analysis (Figure 4D). Then TSLP released by HaCaT cells was assessed by detecting TSLP content in the culture media using ELSIA method. As shown in Figure 4E down-regulation of BRAP in HaCaT cells caused an increase of TSLP that was released by HaCaT cells.

Those data suggest that BRAP homolog deficiency in keratinocytes might cause more production of TSLP during IMQ treatment and thus influence the function of immune cells. The relationship between the function of BRAP homolog in keratinocytes and the changes of cytokines levels during psoriasis-like inflammation process were further discussed in Discussion section.

## 4. Discussion

BRAP was discovered as a novel protein with unknown function in an attempt to search for interacting partners of human Bombesin Receptor Subtype-3 (BRS-3), an orphan receptor^26^. By using a bacteria two-hybrid screening method, BRAP, encoded by *C6orf89*, was found to be a potential partner for BRS-3. However, in this study, we did not further analyze the functional relationship between BRAP and BRS-3 according to the following considerations. First, the interaction between BRAP and BRS-3 was not further verified, either by the yeast two-hybrid experiment using the plasmid expressing full length BRAP, or by co-immunoprecipitation using protein extract of 16HBE14o-, an immortalized human bronchial epithelial cell line that expresses both BRAP and BRS-3^13^. Second, there is no evidence to show the activation effect of BRAP on BRS-3. Finally, BRS-3 is an orphan G-protein coupled receptor (GPCR) belonging to the mammalian bombesin receptor family^27^. GPCRs are seven-transmembrane proteins that are located in the cell membrane. BRAP is a type II membrane protein with a putative transmembrane domain within N-terminal region, a luminal C-domain for an organelle (or an extracellular C-domain for a cell), and an N-terminus for organelle membrane (or a cytoplasmic N-domain for a cell)^28^, ^29^. Its mouse homolog is also predicted to be type II membrane proteins with a putative N-terminal transmembrane (TM) domain, which is similar to BRAP (sequence analyzed by Swiss Model, http://swissmodel.expasy.org/ and AlphaFold structure prediction, https://alphafold.ebi.ac.uk/). Since the immunostaining analysis also showed that most of BRAP signals exist in the cytoplasm in our previous study^29^, we consider BRAP mainly functions in organelles within cytoplasm. While BRS-3 is located in the membrane we did not think the function of BRAP associates with BRS-3 closely.

In our previous studies both BRAP and its mouse homolog were found to be present in various cells including bronchial epithelial cells (BECs), tubular epithelial cells (TECs), lung and kidney fibroblasts, macrophages and neurons. Here we demonstrated that keratinocytes also express BRAP or its mouse homolog, which suggests a role for this protein in keratinocytes. Previously, in order to probe the biological role of BRAP, we established *BC004004^-/-^* mice, in which *BC004004* gene was disrupted so that BRAP homolog was absent in all cells. The organs of *BC004004^-/-^* mice did not show much difference compared with those of wild type controls when examined by histological assays. However, when those mice were induced to develop some diseases, they exhibited alterations in disease phenotypes compared with their wild type controls. Those diseases developed in mice include asthma-like inflammation in lungs induced by oval albumin (OVA) or house dust mites (HDM) (data not published), lung injury and fibrosis induced by bleomycin 12, behavioral changes induced by chronic unpredictable mild stress (CUMS)^13^, renal injury and fibrosis induced either by unilateral ureteral obstruction (UUO) or combined treatment of high fat diet (HFD) and streptozocin (STZ) (data not published). By using those animal disease models we found that BRAP deficiency caused various functional changes in different cell types, which might contribute to alterations in disease phenotypes. In our current study, we observed that* BC004004^-/-^* mice exhibited a more acute onset of skin inflammation induced by IMQ, a potent activator of immune responses that acts as a ligand for TLR7 and TLR8^30^, ^31^. The most severe inflammation occurred on Day 3 and Day 4 and then became alleviated from Day 5 in* BC004004^-/-^*. The inflammatory phenotypes in wild type mice were milder and more sustained, and by the end of treatment (Day 7), the inflammation in wild type mice was more prominent than that of *BC004004^-/-^* mice. The above inflammatory process was accompanied by changes in some cytokine levels within skin or in serum. The initial IMQ application increased both TGF-β1 and IL-23 in *BC004004^-/-^* mice. On Day 4, the inflammation of *BC004004^-/-^* mice was more severe than that of control mice, while the levels of IL-1β, TGF-β1 and IL-17 were also much higher in skin from *BC004004^-/-^*mice. To further investigate the mechanisms underlying the observed phenotype changes in *BC004004^-/-^*, this study primarily focused on exploring the functional alterations in keratinocytes resulting from BRAP homolog deficiency. Our consideration was based on the following observations: first, BRAP was expressed in keratinocytes which are the most abundant cells of epidermis; and second, the first IMQ application could trigger an increase on BRAP homolog expression in skin of wild type mice. However, the question whether functional changes of other cells are more important than that of keratinocytes in the regulation of IMQ triggered immune responses in *BC004004^-/-^* still remains open.

Our data regarding the effect of BRAP deficiency in keratinocytes revealed that the content of TSLP was elevated in skin of *BC004004^-/-^* mice. In addition, down-regulation of BRAP expression in cultured human HaCaT cells by RNAi silencing of *C6orf89* led to more TSLP released into culture media. TSLP is one of the keratinocytes-derived cytokines that mediate the crosstalk between keratinocytes and dendritic cells. It has long been recognized as a master regulator of type 2 immune responses. And its role in asthma and atopic dermatitis (AD), which are typical diseases of type 2 immune responses, has been extensively studied^15^, ^24^, ^25^. Recently, it was also linked to pathogenesis of psoriasis by clinical studies^32^. TSLP level was found to be significantly elevated in the epidermis of untreated psoriasis patients^33^-^36^ . And it was also found to work synergistically with CD40 ligand (CD40L) to promote dendritic cells maturation and the subsequent IL-23 production^33^.

A growing number of studies support that activation of IL-23/Th17 Axis plays a critical role in pathogenesis of psoriasis^17^, ^21^. Since dendritic cells (DCs), which are one of the major sources of IL-23, can be regulated by TSLP^33^, the data of our study provides a possible rational for the altered pattern of pathological changes of psoriasis - like skin lesion in gene knockout mice. The increased release of TSLP by keratinocytes, resulting from BRAP homolog deficiency, may potentially contribute to more production of IL-23 by dendritic cells and thus activated IL-23/Th17 axis during IMQ-induced inflammation. In addition to IL-23, TGF-β1 within skin tissue was also up-regulated after the first IMQ application in *BC004004^-/-^* mice. TGF-β1 has been shown to synergize with IL-23 and IL-1β or other proinflammatory cytokines to differentiate naive T cells into Th17 cells^37^ . TGF-β1 can be secreted by many cells types. RNAi silencing of *C6orf89* in cultured HaCaT cells led to a decrease of TGF-β1 released into culture media as revealed by ELISA (Supplementary Figure 2), which indicates that keratinocytes were not a major source of TGF-β1 within skin tissue of* BC004004^-/-^* mice. The source of TGF-β1 in skin of *BC004004^-/-^* and the mechanism for its up-regulation during IMQ treatment need to be further studied.

Taken together, the present study suggests that BRAP and its homolog participate in the regulation of immune functions of keratinocytes and establishes a foundation for further inquiry into the physiological significance of BRAP in skin.

## Supplementary Material

Supplementary figures.
