# Supplementary material for: Bombesin receptor-activated protein homolog deficiency altered the pattern of pathological changes of psoriasis - like skin lesion in mice
Source: Int J Med Sci. 2024 Jan 1;21(2):357–68. doi: 10.7150/ijms.89492 (PMC10758153; doi:10.7150/ijms.89492)
Supplement: Supplementary file 1 — Supplementary figures. [file ijmsv21p0357s1.pdf]

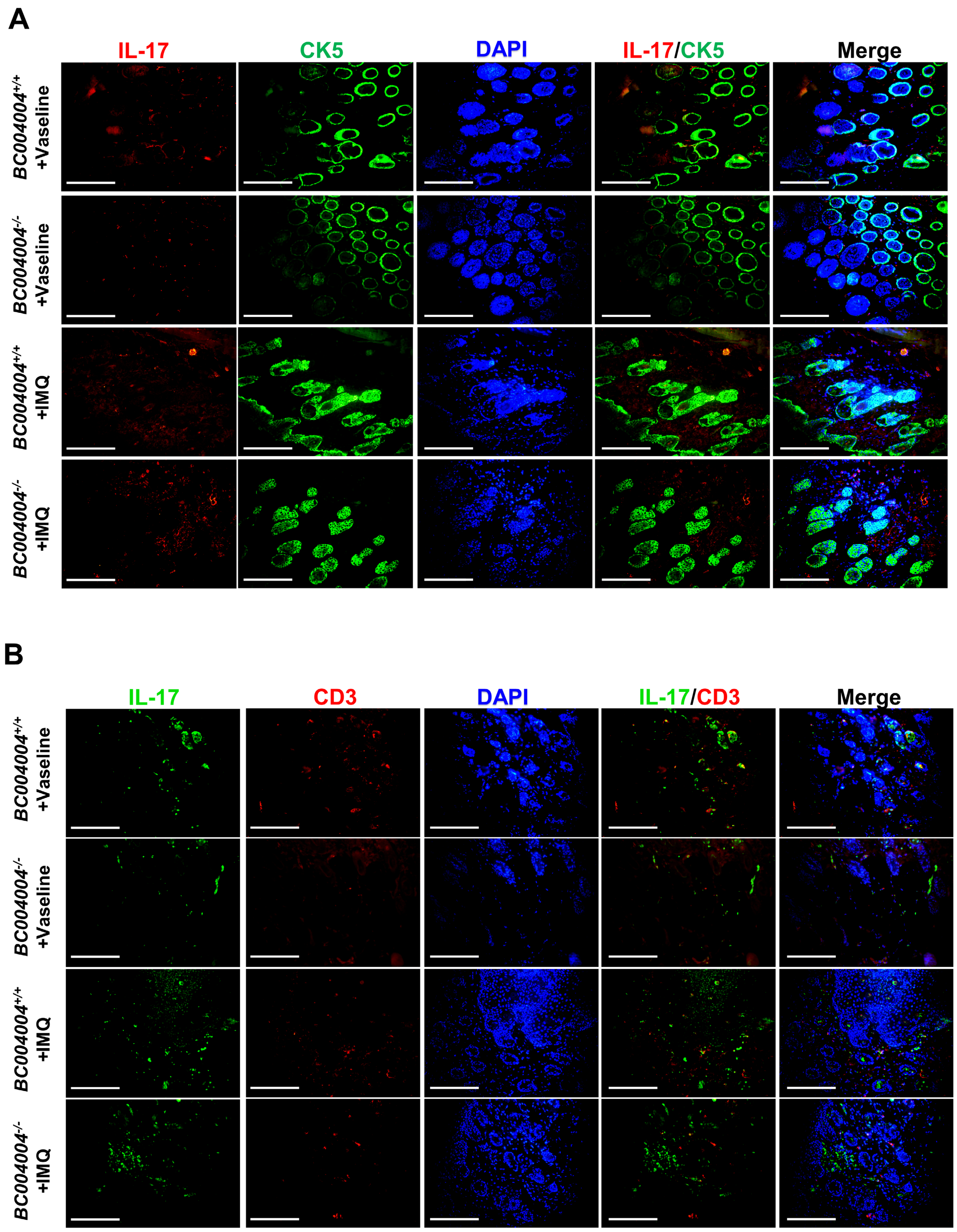

Supplementary Figure 1. Double immunofluorescence analysis of skin tissue sections from IMQ treated mice. Immunostaining with IL-17 antibody was shown as red fluorescence. Immunostaining using

antibodies against CD3 or CK5 was shown as green fluorescence. Nuclei of the cells were localized by DAPI (blue)(magnification: 200×); scale bar = 100 μm.

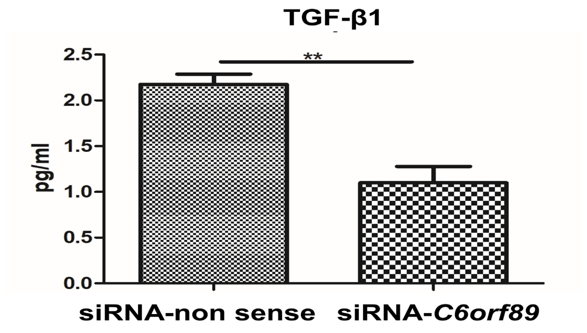

Supplumentary Figure 2. ELISA analysis of TGF-β1 content in the culture media of HaCaT cells with *C6orf89* expression silenced by siRNA transfection.
